# Who Is Serving Us? Food Safety Rules Compliance Among Brazilian Food Truck Vendors

**DOI:** 10.3390/ijerph15122807

**Published:** 2018-12-10

**Authors:** Lígia Isoni Auad, Verônica Cortez Ginani, Eliana dos Santos Leandro, Aline Costa Santos Nunes, Luiz Roberto Pires Domingues Junior, Renata Puppin Zandonadi

**Affiliations:** 1 Department of Nutrition, Faculty of Health Sciences, University of Brasília (UnB), Campus Darcy Ribeiro, Asa Norte, Brasilia DF 70910-900, Brazil; vcginani@gmail.com (V.C.G.); elisanleandro@yahoo.com.br (E.d.S.L.); renatapz@yahoo.com.br (R.P.Z.); 2 Department of Pharmacy, Faculty of Health Sciences, University of Brasília (UnB), Campus Darcy Ribeiro, Asa Norte, Brasilia DF 70910-900, Brazil; alinecostasn@gmail.com; 3 Department of Mechanical Engineering, University of Brasília (UnB), Campus Gama, Brasilia DF 72444-210, Brazil; luizr.dominguesjr@gmail.com

**Keywords:** food truck, food safety, vendor, Brazil

## Abstract

The rise of food trucks as an eating out option requires knowledge of this sector. Balancing the reality of the food truck sector with access to safe food should guide actions and public policies to cater to its peculiarities. Therefore, this study aimed to characterize the Brazilian food truck vendors’ profile regarding their socioeconomic status and compliance with food safety rules. From the 118 food truck vendors registered in the Brazilian Federal District, 30% (*n* = 35) participated in the study. We conducted structured interviews from December 2017 to April 2018. We ranked compliance levels according to a five-point Likert scale based on calculated compliance scores. The interviews revealed that food truck vendors were mostly married males, who had completed at least a tertiary education level, and wanted to start up their own businesses. The compliance levels depict good compliance with food safety rules (overall compliance (OC)-score = 0.69, on a 0 to 1 scale). The food trucks assessed in this study distinguished themselves from the street food and food retail sectors due to their operational structure and the complexity of food production processes. Those particular features should be considered to ensure adequate and effective sanitary control and inspections, as well as to reduce the probability of microbial growth and food contamination and the consequent risk of foodborne illnesses.

## 1. Introduction

The term “street food” (SF) describes a wide range of “ready-to-eat foods and beverages prepared and/or sold by vendors or hawkers especially in the streets and other similar places” [1]. In developing countries, especially in urban areas, street food plays a significant role in the socioeconomic and nutritional spheres for low-and-middle-income vendors and consumers, generating employment and constituting a ready and inexpensive of nutrition to the diet of locals [2,3]. 

The processes of globalization and urbanization have been changing the feeding profile of the population [4]. It is noteworthy that meals prepared at home have been increasingly replaced by fast food meals when eating out in places of easy access and with fast food services like food trucks (FTs) [5]. In the past few years, the emerging industry of gourmet FTs has been revolutionizing the street food scene. The Brazilian FT industry has been booming, especially over the last four years, also due to the influence of the American boom of FTs. According to statistics collected by Serviço Brasileiro de Apoio às Micro e Pequenas Empresas (Brazilian Micro and Small Business Support Service) SEBRAE [6], the annual revenue of FTs in Brazil in 2014 was 140 billion reais (about 630 billion USD). FTs have also become an increasingly popular trend in the Brazilian Federal District in the last few years, offering an alternative source of high-quality food at more affordable prices than those offered by full-service restaurants. Despite its popularity, there are no studies concerning FTs nor FT vendors (FTVs) in Brazil.

As FTs have proliferated in big cities across the world, business and health regulations have become inevitable. Policies and regulations have played a major role in recognizing FTs as a formal sector, as well as ensuring food safety and protecting consumer health These regulations are usually comprised of distinct policy areas—including economic activity, public space, and public health—which have a particular approach in each country and locality. Regardless of their variation, the FT regulatory frameworks usually consider the use of a commissary kitchen for food storage and preparation and the transportation process to selling points. The complexity of menu items, including the high number of ingredients and processes involved in food processing, requires special attention to the food process chain regarding time and temperature control. Therefore, the challenges for process control bring FTs closer to brick-and-mortar establishments. On the other hand, the selling points and the environmental conditions in which FT trade is practiced is similar to that found in the street food vending sector.

In the Brazilian Federal District, FT activity is regulated by the local laws no. 5.627 [7] and no. 11 [8], both enacted in 2016, which cover aspects of the utilization of public property, including time constraints, parking restrictions, and geographic limitations related to commercial zones; and public health and safety, which encompasses aspects related to sanitation and food safety. FTs in the Brazilian Federal District are also subjected to the same health and safety regulations as other food services providers, such as brick-and-mortar restaurants, meaning that they must comply with national standards established by Board Resolution 216 (RDC 216) [9].

Although FTs provide a convenient and unique gastronomic experience and positively impact the local and national economies [10,11], the street vending activity is considered an undesirable part of the informal sector, especially due to the associated risks concerning food safety. SFs have been linked to approximately 70% of disease outbreaks [12] and are also considered potential sources of enteropathogens [13]. Thus, the importance of compliance to food safety regulatory requirements by FT vendors to prevent or mitigate a serious danger to public health cannot be overemphasized. However, there are neither studies concerning FTs nor the actual conditions and dynamics of this sector, despite their ubiquity in metropolitan areas all over the world, including in the Brazilian Federal District.

Since the existing institutional and legislative frameworks have an impact on food safety among food vendors, this study aimed to characterize the Brazilian food truck vendors’ (FTVs) profile regarding their socioeconomic status and compliance with food safety rules. As in other developing countries, the FT sector in Brazil is characterized by a weak capacity to monitor and enforce legally defined minimum hygiene standards. Therefore, the findings of this study will be of great importance to public health authorities, policy makers, and implementing bodies to ensure compliance with food safety rules among FTVs in order to improve on the implementation of the existing food safety laws and, ultimately, to ensure consumers’ access to safe street food.

## 2. Materials and Methods 

**Figure 1:**
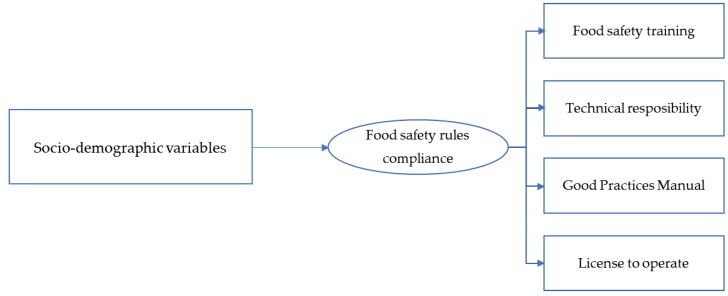
Conceptual framework of the study.

This is a cross-sectional, exploratory, and descriptive study carried out in the Federal District of Brazil, from December 2017 to April 2018. We used a convenient sample of 35 FTVs registered in the Brazilian Federal District Health Surveillance Agency (*n* = 118), since the local and frequency of FT activity is not standardized nor previously informed. The exclusion criterion was the FTV not willing to participate in the interview. We used a structured multiple-response questionnaire (supplementary file 1) comprised of 19 items that contained questions about the vendors’ socioeconomic conditions (gender, age, level of education, monthly income); information about their work in the FT (including experience in food services, how long their business had been operating, and the reason why they started a FT business); and the food safety rules compliance, namely training in food safety, the presence of a certified person in charge, the possession of the Good Practices Manual, and the possession of a license to operate the FT business, required by Brazilian national and local legislations [7,8,9]. Figure 1 illustrates the conceptual framework of this study.

We calculated a compliance score (C-score) for each food safety rule, represented by the equation below:

(1) $$\mathrm{Compliance}\;\mathrm{Score}\;\left(\text{C-score}\right)=\frac{N_{c}}{T}$$

N_c_ = Number of FTVs complying with a particular food safety rule;

T = Total number of FTVs.

Compliance levels of food safety rules were based on a five-point Likert scale and evaluated according to the recommendations of Money et al. (2014) [14], in which a compliance score of 0.80–1.00 indicates a very good compliance level, 0.60–0.80 indicates a good compliance level, 0.40–0.60 indicates an average compliance level, 0.20–0.40 corresponds to a poor compliance level, and 0.20–0.0 indicates a very poor compliance level.

We calculated the overall C-score (OC-score) separately, according to the equation below, and it was based on the C-scores obtained for each food safety rule. This represents the overall average compliance to all food safety rules assessed in the study.

(2) $$\mathrm{Overall}\;\mathrm{Compliance}\;\mathrm{Score}\;\left(\text{OC-score}\right)=\frac{\sum_{n}^{i}{\text{C-score}}_{i}}{n}$$

C-score_*i*_ = Compliance score of the *i*th food safety rule

*n* = *n*th food safety rule or principle

All interviews were conducted in Brazilian Portuguese and performed at the FT vehicle during the operation shift of the food vendor. Data were tabulated using Microsoft Office Excel 2013 (Microsoft, Redmond, WA, USA) and analyzed using descriptive statistics.

The ethical and methodological aspects of this study had the approval by the Ethics Commission of the University of Brasília (UnB), registered under number CEP no. 2.178.214. We assured anonymity and confidentiality of participants throughout the study.

## 3. Results

We performed the present study with vendors from FTs that were usually parked in specific locations during the night in clusters of between 6 to 10 vehicles, offering different menu options. Among the ready-to-eat food options FTVs were selling, 40% offered hot and cold sandwiches, while 31% offered pizza and pasta, and the other 29% offered international and regional cuisine. Despite serving traditional meals, such as sandwiches and hot dogs, FTs provided all food options with a modern/sophisticated touch to revisit the traditional dishes, including gourmet ingredients and special gravies and sauces. Since these FTs tend to serve higher quality food at higher prices than more traditional mobile vendors, they are considered ‘gourmet’ [15]. Gourmet FTs tend to operate in urban areas, satisfying the demanding taste of their clients and using specific cooking techniques and, consequently, a higher number of ingredients and food preparation processes.

From a total of 118 FTVs registered in the Brazilian Federal District Health Surveillance, 63 (53%) were found and invited to participate in the interview, and 35 (30%) agreed to participate. The interviews took 20–25 min to complete. 

Table 1 presents the socio-demographic characteristics of the FTVs. Most of the 35 FTVs interviewed (average age 41 years ± 10.45, range 20–75) were male (77%), white (69%), Brazilian (94%), married (60%), with two children (*n* = 13; 37%), and had completed at least a tertiary level of education (57%).

From the 35 FT vendors interviewed, 11% (*n* = 4) reported a lack of employment as a reason to start a FT business. However, 40% of FT vendors (*n* = 14) mentioned that the opportunity to have their own business was the main influencing factor. The average income for over a third of the vendors was at least 17-fold higher than the national average income (approximately US$ 6090). Almost half of the vendors reported that they had invested at least R$ 50,000 (US$ 12,000) to enter the FT niche.

Most vendors (*n* = 33; 94%) reported using a commissary kitchen as a home base of operations for preparing and storing food, as well as to park their vehicles, and employing at least one assistant in the commissaries. Staff were also employed at the FTs to assist with daily routine. When asked about the recruitment process, only four (11%) vendors reported using employee referral during the hiring process. Eight vendors (23%) reported taking into consideration the former experience of the candidate in the food business, while nine FTVs (26%) declared that they hired family members.

A quarter of the vendors had been working in a FT for less than one year, while more than a half (*n* = 19) had been operating a FT for at least two years. Sixty-two percent (*n* = 22) of FTVs reported selling an average of 50 meals daily, while 31% (*n* = 11) sold between 50 and 100 meals per day, and only 6% (*n* = 2) sold more than 100 meals per day. The majority of owners (*n* = 21; 60%) had previous experience in the food service business as owners of brick-and-mortar establishments or as fast food restaurant managers.

**Figure 2:**
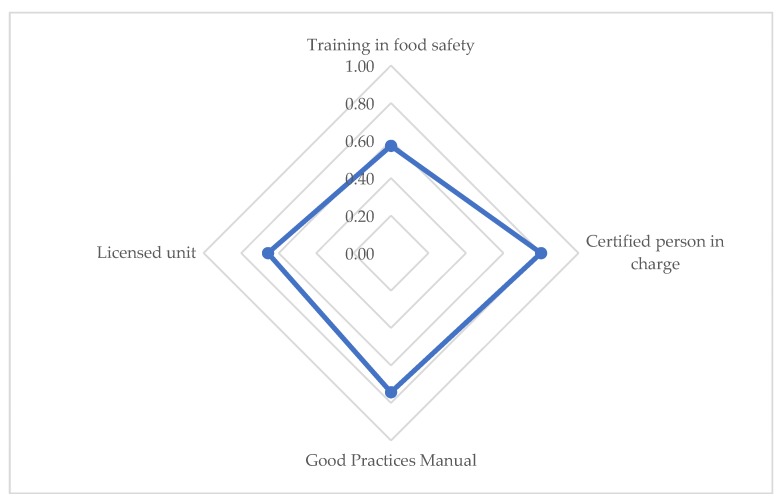
Compliance scores of food safety rules.

Figure 2 shows the compliance levels of food safety rules among FTVs. We verified marginally very good compliance for a certified person in charge (C-score = 0.80). We observed good compliance with the possession of a Good Practices Manual (C-score = 0.74) and the licenses and permits needed to run a FT (C-score = 0.66). Also, we verified average compliance for training in food safety (C-score = 0.57).

## 4. Discussion

In Brazil, the National Health Surveillance Agency (Agência Nacional de Vigilância Sanitária—Anvisa) is responsible for the regulation, control, and inspection of products and services involving public health risks—which include the food sector. These actions are shared with other ministries and integrate the National System of Sanitary Surveillance (Sistema Nacional de Vigilância Sanitária—SNSV). The SNSV devolves on different tiers of surveillance authorities: federal, state, and local. Due to its public nature and the highly external scope of its field of action, as well as to the heterogeneity of the Brazilian regions, the SNSV requires federal coordination to increase local and regional cooperation [16].

Considering the continuous improvement of sanitary control, the harmonization of the sanitary inspections, and the elaboration of general hygienic-sanitary requirements for food services, in 2014 the Anvisa Collegiate Board of Directors approved the Technical Regulation for Good Practices in Food Services [9]. This federal regulation applies to all establishments where food is handled, prepared, stored, distributed, transported, and/or exposed to sale or service—including restaurants, canteens, buffets, industrial and institutional kitchens, snack bars, bakeries, delicatessens, rotisseries, and similar forms of establishments.

**Figure 3:**
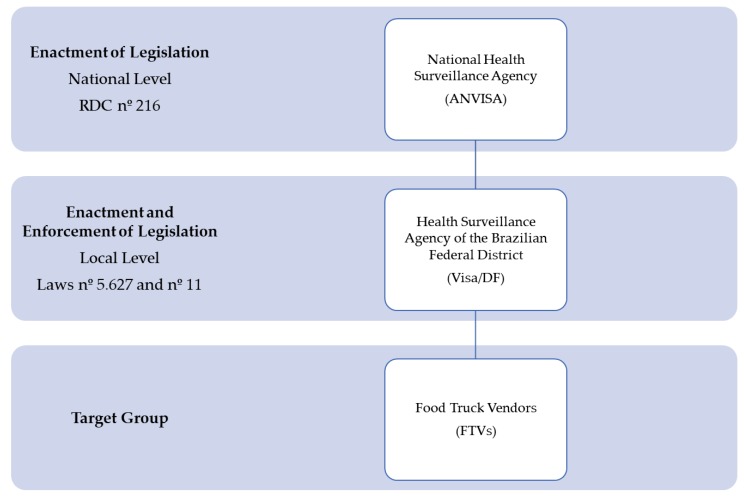
National and local legislation of food trucks in the Brazilian Federal District. Anvisa: Agência Nacional de Vigilância Sanitária (National Health Surveillance Agency); DF: Distrito Federal (Federal District); FTVs: food truck vendors.

FTs in the Brazilian Federal District must comply with RDC 216 [9], since they perform food handling and selling activities in the vehicles and in the commissaries. In addition, they must comply with the local laws no. 5.627 [7] and no. 11 [8], both enacted in 2016, the former which regulates FT activity and the latter which defines the registration and inspection procedures for the regularization of FTs (Figure 3). Both national and local legislations establish common food safety recommendations to which FTs in the Brazilian Federal District must adhere, including food safety training, the presence of a certified person in charge, the possession of the Good Practices Manual, and the licenses and permits to run the business, all of which were assessed in this study.

FTs must comply with local ordinances. However, there is a lack of consensus on FT classification, which leads to regulatory divergence and, consequently, to an unharmonized inspection system. In Brazil, FT legislation has only been established in a few states and municipalities. On the one hand, São Paulo, Rio de Janeiro, and Porto Alegre state capitals have applied their existing regulations of the SF sector to FTs. Therefore, FTs in those locations are inserted in the SF activity. As earlier mentioned, the Brazilian Federal District, on the other hand, has a specific regulation for FTs, which refers to the national legislation for Good Practices in food services and, therefore, states that FTs are inserted in the same category as brick-and-mortar restaurants.

The dissensus concerning FT classification is not restricted to Brazil. For the Workers’ Compensation Insurance Rating Bureau of California (WCIRBC) [17], mobile food vending—including FTs—should be assigned to appropriate classification based on the operations performed. According to the WCIRBC, since mobile food vendors typically operate at public and industrial sites, on a temporary or intermittent basis, preparing and serving hot and cold foods, they compete with restaurants and should be classified as such. Also, according to Williams [18], FTs are an itinerant miniature commercial kitchen that must meet the same sanitation requirements as a brick-and-mortar restaurant. At the same time, for the Department of Health and Mental Hygiene of New York City, FTs are classified as mobile food vending units and, therefore, must operate in full compliance with existing rules and regulations for mobile food vending. For that reason, the mobile food industry is not a distinct nor clearly identifiable industry for which a unique classification can be established and, therefore, a new classification should not be set [17].

Due to a lack of international consensus regarding the classification of FTs and the scarcity of literature concerning the assessment of the food safety practices and conditions of these vehicles, the comparison baseline for the findings of this research considered studies performed in the SF and food retail sectors.

The sociodemographic results of this study revealed that most of the FTVs were married males who had completed at least a tertiary level of education. We also observed a very low rate of immigrants among the FTVs interviewed (6%). The FT movement was partly spurred by the 2008 recession as an alternative form of job for many highly qualified but unemployed chefs. Therefore, contrary to the food service sector in which vendors often suffer from a lack of professional skills and resources [19,20,21], an innovative entrepreneurial class of largely white, native-born, culinary-school-trained professionals seeking new business opportunities leads the FT generation [22], a description that concurs with the findings of this study. Additionally, the significant growth of culinary courses and schools in Brazil is also responsible for the expansion of the service and hospitality sectors in the country [23]. However, these findings are in disagreement with the previously established literature concerning the SF sector, in which street food vendors have mainly been women [24,25,26,27,28,29,30,31] with low educational level [14,32,33,34], and often composed of immigrants [22].

In many developing countries, the street food trade is considered a part of the informal economy and represents a regular source of income and employment for urban populations. Also, the street vending business can represent an alternative to the saturated formal sector, ensuring a source of supplemental income from other activities, as earlier studies have revealed [35,36]. According to previous studies, one of the main motivating factors to engage in street vending activity is a lack of employment [37,38,39]. However, the expansion of the street vending sector is not only due to unemployment. Although some vendors reported a lack of employment as a reason to start a FT business, the majority of them mentioned that the opportunity to have their own business was the main influencing factor. A study performed in China [40] found that street vendors have diverse motivations for participating in street vending, including unemployment, the low quality of waged jobs, rural poverty, and also the desire to achieve autonomy and flexibility while earning a living. Self-employment may indeed enable vendors to find a balance between their personal and professional lives. Additionally, FTs have financial and logistic advantages in comparison to brick-and-mortar restaurants which attract budding entrepreneurs, including low start-up costs, fewer permits and operational expenses, lower maintenance costs, easier customer outreach, and versatile site selection.

Over a third of the vendors in the current study reported an average monthly income above 24 times the minimum wage (Brazilian minimum wage is R$ 954 per month—about US$ 290). Given that the Brazilian Institute of Geography and Statistics [41] puts the average per capita household monthly income in Brazil at R$ 1268 (approximately US$ 306), the average income of these vendors is at least 17-fold higher than the national average income. The FT business in the Federal District can, therefore, be considered a particularly lucrative activity. Also, a high initial investment is required to start a FT business, which includes the purchase of the truck, equipment, food ingredients, and other expenses. As expected, half of the vendors reported to had invested at least R$ 50,000 (US$ 12,000) to enter the FT sector, and almost all owned the FT they worked at (*n* = 34). Contrarily, SF activity in low- and middle-income countries constitutes a major source of income and employment for the poor, with vendors usually experiencing low average earnings, which makes street vending in those locations an unprofitable activity [21,35,36,37,38,40,42,43].

Besides the high initial investment, a FT operation also requires significant on-going monthly costs with a commissary kitchen and employees. It is noteworthy to mention that the local FT law no. 5.627 of the Brazilian Federal District requires the use of commissaries, which are subjected to inspections and must maintain local health standards. In this study, 26% of the FTVs reported employing family members in the FT or the commissaries. Relatives helped at the place of sale and at home preparing food or buying ingredients. The presence of family members is also a common feature in the street food sector, as previous studies have revealed [37,38,44,45,46]. SF vending is also a family survival strategy, since unpaid family labor allows higher incomes to vendors that otherwise would not be possible if they had done the same work alone or utilized paid labor [47]. 

Food safety training (C-score = 0.57) was among one of the four food safety rules assessed in this study, which almost two-thirds of the FTVs reported having (*n* = 21; 60%). This finding is in disagreement with studies conducted in food service establishments [48,49] and in the street vending sector in other developing countries, including Togo, Nigeria, Vietnam, Malaysia, and the Philippines [26,27,50,51], where the vast majority of vendors did not have any food safety training. However, it concurs with a study performed in the Brazilian food retail sector in which head chefs and managers of hotels’ restaurants showed good performance in food safety due to their participation in training courses, and most of them had certificates [52]. Despite the fact that knowledge does not necessarily translate into changes in behavior [30], the findings of international literature indicate that food hygiene training, along with adequate food handling practices, is a crucial element in the control of foodborne illnesses [38,53,54,55]. The previous experience in the food business that most vendors reported to have, as well as their high educational level, may also positively affect the level of food safety knowledge, since the connection between educational level and personal hygiene has been previously highlighted [32]. The level of workers’ formal schooling, the participation in training courses, and the positive experience in the professional field are factors that contribute towards ensuring food safety [56].

According to the RDC 216 [9], technical responsibility should be undertaken by a certified owner or a designated employee with Good Practices training. In our study, 83% of the FTVs (*n* = 29) reported the presence of a certified person in charge (C-score = 0.80). From those 29 vendors, 19 (65%) cited that the designated person in charge was a registered dietitian/nutritionist and six (21%) reported that they themselves had the technical responsibility. Besides complying with the national legislation, the assistance of professionals with technical knowledge is important during the implementation and maintenance of Good Practices and may positively impact on employee’s food handling behavior to reduce the risk of foodborne illnesses, as some studies have demonstrated [57,58].

According to the local law no. 11 [8], to obtain the required health permit to operate, FTs must present a Good Practices Manual comprised of a list of ingredients and raw materials used to prepare menu items and their respective flow process charts, as well as the FT and the commissary kitchen layouts. In the current study, the possession of a Good Practices Manual (C-score = 0.74) was reported by more than three-quarters of FTVs (77%). This finding is in disagreement with a study carried out in self-services restaurants in the city of São Paulo, Brazil, that found that the Good Practices Manual was not available in most of the establishments surveyed [59]. Besides the health permit, Brazilian FT owners must meet specific requirements to operate their business, including business permits and commercial vehicle registration, on-site inspections of the FT and the commissary kitchen issued from the local Health Surveillance Agency, and fire safety approvals from the local Fire Department. Many owners in our study (69%) reported having the licenses and permits needed to run a FT (C-score = 0.66). This finding disagrees with earlier reports of Choudhury et al. [38] and Annan-Prah et al. [60], where 54% and none of the vendors had a license to operate their business, respectively. 

Overall, the findings of this study revealed good compliance concerning the food safety rules (OC-score = 0.69) among Brazilian FTVs. The levels are higher than the results previously obtained by the studies mentioned above, both in the SF and food retail sectors. The adherence averages in this study may be larger in those found in other surveys due to the FTVs’ higher educational levels, training, and previous experience in the food service sector, which may also indicate familiarity with food safety requirements.

This study also revealed that a lack of consensus regarding FT classification implies having distinct regulations and, consequently, heterogeneous food safety standards, which weaken their implementation and hinder enforcement capacity. The FT sector may benefit from a national approach to food safety legislation, including the food safety framework from the SF and food retail sectors, in order to reduce cross-jurisdictional discrepancies in regulation [61]. Moreover, this would facilitate the balance of the many competing interests involving the regulation of the FT industry and ensure harmonization of its sanitary inspections and control. 

Considering the exponential increase in the consumption of food prepared outside the home, there is a greater risk exposure to poor hygiene in commercial food service settings [62] and, consequently, to foodborne illnesses. Therefore, studies in the FT sector are required in order to shed light on food preparation and selling conditions. This research is part of a larger study, which is currently in progress. The hygienic-sanitary practices and conditions of FTs of the Federal District will be assessed by the application of an evaluation instrument, along with the collection of food and water samples for microbiological examination, to determine if food safety knowledge is positively influencing behavior and practices.

### Limitations

There are several potential limitations in this study. Firstly, the small sample size and its convenient nature mean that the data presented here may not be representative of FTs in the Federal District or other regions of Brazil. During this study, 63 potentially eligible FTs were identified and invited to participate in the interview. Despite the use of a written-consent approach and the awareness of the anonymity and confidentiality of the study by the participants, we observed a high refusal rate (46%; 29 FTVs). Repeated attempts, aiming to increase the sample of FTVs, were unsuccessful. Among the most prevalent reasons for refusing participation in the study were a time constraint, lack of interest in the research, fear of identification, and fear of confidential information leakage. Due to the pilot nature of this study, an increase in the number of participants should be considered in future iterations. The second limitation lies in its voluntary nature, since non-compliant FTs may have refused to participate in this study. Another limitation of this research is that the interviews had to be held on the street and during the FTV operation shift, which implied a time limit (the interviews took 20–25 min to complete) and a crowded and noisy environment. Despite these limitations, the interviews with the vendors produced consistent information, as presented in this study.

## 5. Conclusions

FT vending is an integral part of a country’s gastronomic and cultural identity. The findings of this study reveal that FTs are a distinct sector in the Brazilian foodservice field due to the unique socioeconomic profile of their vendors, who are mainly highly educated, well qualified, and possess a favorable financial condition. The current findings also indicate that most FTVs met the national and local legal food safety requirements. Although a legislative framework for FTs exists in some Brazilian states and in the Federal District, the FT sector would benefit from the development of a national law that encompasses food safety frameworks of the street food and the food retail sectors to ensure harmonization of the sanitary inspections and control, as well as to reduce the probability of microbial growth and food contamination and the consequent risk of foodborne illnesses. 

## Figures and Tables

**Table 1:** Socio-demographic profile of food truck vendors studied in the Federal District, Brazil.

Characteristic Evaluated	Response	Frequency (*n* = 35)	Percentage (%)
Age (years)	≤20	2	5.7
	21–30	7	20.0
	31–40	15	42.8
	>40	11	31.4
Gender	Male	27	77.1
	Female	8	22.9
Color	White	24	68.6
	Black	11	31.4
Nationality	Brazilian	33	94.3
	Other	2	5.7
Marital status	Married	20	57.1
	Single	12	34.3
	Divorced	3	8.6
Children	0	9	25.7
	1	8	22.9
	2	13	37.2
	3	3	8.6
	4	1	2.8
	5	1	2.8
Level of education	Secondary	15	42.9
	Tertiary and above	20	57.1
Reason to start a food truck business	Unemployment	4	11.4
	Opportunity of starting my own business	14	40.0
	Feeling connected to the food truck business	5	14.3
	Food truck is my side job	1	2.9
	Formal markets are saturated	1	2.9
	Other	10	28.5
Number of meals sold per day	≤50	22	62.8
	50–100	11	31.4
	100–150	1	2.9
	>150	1	2.9
Monthly income (R$; minimum wage)	1–4	5	14.3
	5–9	8	22.8
	10–14	5	14.3
	15–19	4	11.4
	20–24	1	2.9
	>24	12	34.3
Investment in the food truck (R$)	Rental	1	2.9
	10k–30k	6	17.1
	30k–50k	11	31.4
	50k–70k	3	8.6
	70k–90k	7	20.0
	>90k	7	20.0
Previous experience in food services	Yes	22	62.9
	No	13	37.1

## Supplementary Materials

The following are available online at http://www.mdpi.com/1660-4601/15/12/2807/s1, File 1: Food truck vendor questionnaire.
