# Supplementary material for: Who Is Serving Us? Food Safety Rules Compliance Among Brazilian Food Truck Vendors
Source: Int J Environ Res Public Health. 2018 Dec 10;15(12):2807. doi: 10.3390/ijerph15122807 (PMC6313619; doi:10.3390/ijerph15122807)
Supplement: Supplementary file 1 [file ijerph-15-02807-s001.pdf]

FOOD TRUCK VENDOR QUESTIONNAIRE

**Number:**	**Year:**
**Food Truck:**	
**Owner:**	
**Email/Phone:**	

1. Gender

( ) Male
( ) Female

2. Color

( ) White
( ) Black
( ) Yellow
( ) Red

3. Nationality

( ) Brazilian
( ) Other

4. Age

( ) ≤20
( ) 21-30
( ) 31-40
( ) >40

5. Marital Status

( ) Married
( ) Single
( ) Divorced
( ) Widowed

6. Children

( ) 0
( ) 1
( ) 2
( ) 3
( ) 4
( ) ≥5

7. Level of Education

( ) Primary (Elementary School)
( ) Secondary (High School)
( ) Tertiary (Graduation)
( ) Quaternary (Post-graduation)

8. Reason to start a food truck business

( ) Unemployment
( ) Opportunity of starting my own business
( ) Feeling connected to the food truck business
( ) Food truck is my side job

( ) Formal markets are saturated
( ) Other

9. Number of meals sold (per day)

( ) ≤50
( ) 50-100
( ) 100-150
( ) >150

10. Monthly income (R$; minimum wage)

( ) 1-4
( ) 5-9
( ) 10-14
( ) 15-19
( ) 20-24
( ) >24

11. Investment in the food truck (R$)

( ) Rental
( ) 10k-30k
( ) 30k-50k
( ) 50k-70k
( ) 70k-90k
( ) >90k

12. Previous experience in food services

( ) Yes
( ) No
If yes, please specify your previous experience in the foodservice business: ___________________________________

13. Training in food safety/food hygiene

( ) Yes
( ) No
If yes, have you received training in the last 6 months?
( ) Yes
( ) No

14. Employee(s) in the food truck

( ) 0
( ) 1
( ) 2
( ) 3
( ) 4
( ) >5

15. Employee(s) in the commissary kitchen

( ) 0
( ) 1
( ) 2
( ) 3
( ) 4
( ) >5

16. Recruitment and hiring process
( ) Previous experience in food trucks<br>( ) Previous experience in the foodservice business<br>( ) Family member<br>( ) Employee referral<br>( ) Other
**17. Certified person in charge for the food handling practices**
( ) Yes<br>( ) No<br>If yes, please specify: ___________________________________
**18. Best Practices and Standard Operating Procedures Manual**
( ) Yes<br>( ) No<br>( ) Under implementation
**19. Licensed food truck**
( ) Yes<br>( ) No<br>( ) Under implementation
